# Multidrug-resistant *Salmonella* Typhimurium Infection from Milk Contaminated after Pasteurization

**DOI:** 10.3201/eid1005.030484

**Published:** 2004-05

**Authors:** Sonja J. Olsen, Michelle Ying, Meghan F. Davis, Marshall Deasy, Ben Holland, Larry Iampietro, C. Michael Baysinger, Frances Sassano, Lewis D. Polk, Betty Gormley, Mary Jane Hung, Keith Pilot, Maria Orsini, Susan Van Duyne, Shelley Rankin, Carol Genese, Eddy A. Bresnitz, Joseph Smucker, Maria Moll, Jeremy Sobel

**Affiliations:** * Centers for Disease Control and Prevention, Atlanta, GA, USA; † Pennsylvania Department of Health, Harrisburg, Pennsylvania, USA; ‡ Montgomery County Department of Health, Norristown, Pennsylvania, USA; § Bucks County Department of Health, Doylestown, Pennsylvania, USA; ¶ Gloucester County Health Department, Turnersville, New Jersey, USA; # New Jersey State Department of Health and Senior Services, Trenton, New Jersey, USA; ** University of Pennsylvania, Philadelphia, Pennsylvania, USA; †† U.S. Food and Drug Administration, Washington, D.C., USA

**Keywords:** *Salmonella*, disease outbreak, drug resistance, milk, food contamination

## Abstract

An outbreak of multidrug-resistant *Salmonella* *enterica* serotype Typhimurium infections occurred in Pennsylvania and New Jersey. A case-control study implicated pasteurized milk from a dairy, and an inspection indicated the potential for contamination after pasteurization. Dairy cattle are the likely reservoir, and milk may be an important vehicle of *Salmonella* transmission to humans.

Pasteurization, or heat treatment, of milk is an important milestone in public health that contributed to dramatic declines in many infectious diseases. Despite the important public health gains achieved, outbreaks associated with pasteurized milk continue to occur (*1*–*3*). We describe a recent outbreak associated with pasteurized milk.

## The Study

On April 13, 2000, the Pennsylvania State Department of Health notified the Centers for Disease Control and Prevention (CDC) of an increase in *Salmonella* *enterica* subspecies *enterica* serotype Typhimurium. Active surveillance for *Salmonella* group B and serotype Typhimurium was initiated, and health officials in Maryland, Delaware, New Jersey, and New York were notified. Isolates were sent to public health laboratories for confirmation and serotyping and to New Jersey and CDC for antimicrobial susceptibility testing by MIC with broth microdilution and molecular subtyping by pulsed-field gel electrophoresis (PFGE) and phage typing (*4*,*5*). To identify and compare isolates from cows during the time of the outbreak, we contacted personnel at the *Salmonella* Reference Center at the University of Pennsylvania.

Stool samples from 93 persons yielded *S.* Typhimurium (76 in Pennsylvania and 17 in New Jersey). Dates of illness onset ranged from March 6 to April 19 (Figure). The median age of patients was 9 years (range 3 months–88 years), and 51 (55%) were male. PFGE was performed on 44 of 93 isolates from patients. Of the 44 isolates, 26 were pattern A, 1 pattern B, 5 pattern C, 7 pattern D, and 1 each for pattern E, F, G, H, and I. The three dominant patterns (A, C, and D) formed a complex of highly related strains (1 band difference, 1 band shift, or both). We defined the 38 isolates with patterns A, C, and D as outbreak-related strains. The outbreak-related strains were unique when compared to the other 3,469 *S.* Typhimurium PFGE patterns in the PulseNet database (CDC, Atlanta, GA). Isolates identified as outbreak-related strains were all phage type 21. Of the 16 isolates tested for antimicrobial resistance, 12 (7A, 1B, 1C, 3D) were resistant to ampicillin, kanamycin, streptomycin, sulfamethoxazole, and tetracycline (AKSSuT), 3 (1A, 2C) were AKSSu, and 1 (A) was ASSu resistant. Two of the three *S.* Typhimurium isolates obtained from dairy cows during the same time period were also outbreak-related strains.

**Figure:**
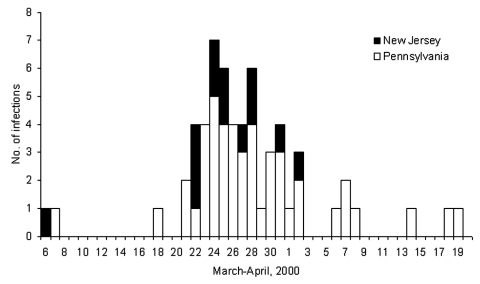
Dates of illness onset among persons with *Salmonella enterica* subspecies *enterica* serotype Typhimurium infection, Pennsylvania and New Jersey, March-April, 2000

We conducted 11 interviews with patients who had recent onset of disease; during these interviews, infection through milk consumption became a leading hypothesis. In the case-control study, a case was defined as an outbreak-related isolate of *S.* Typhimurium in a resident of Pennsylvania or New Jersey with illness onset between March 1 and April 21. If more than one person in a household met the case definition, we interviewed the person with earliest onset. Two controls per case were selected by random digit dialing and matched to patients by area code and three-digit telephone prefix and age group. Using a questionnaire administered over the telephone, we asked about exposures in the 5 days before patient’s illness onset. Parents responded for children.

We interviewed 23 (61%) of the 38 eligible case-patients and 37 controls; they did not differ with respect to age or sex (median age 5 years vs. 5 years, p = 0.8; percent male: 70% vs. 54%, p = 0.2). Dates of diarrhea onset were from March 20 to April 19. Twenty-two (96%) patients reported diarrhea, 17 (74%) bloody diarrhea, 20 (87%) abdominal cramping, 16 (70%) fever, and 8 (35%) vomiting. In addition, 6 patients (26%) were hospitalized, and 9 (39%) were treated with antimicrobial drugs (amoxicillin [2 patients], ciprofloxacin [2 patients], sulfamethoxazole [1 patient], unknown agent [4 patients]).

Ill persons were 22 times more likely to drink milk from dairy plant X than controls (20 of 23 patients vs. 13 of 37 controls, matched odds ratio = 23, 95% confidence interval = 2.7 to 184.5). Odds of infection increased with drinking larger amounts of milk from dairy plant X (p = 0.0008). Other risk factors, including handing reptiles or consuming chicken, undercooked eggs, sprouts, or unpasteurized milk or juice were not associated with illness. Although Philadelphia patients were excluded from the case-control study for logistic reasons, seven were residents or employees of an independent living facility where more than 80% of the milk received was from dairy plant X.

During the last 2 weeks of April, state and federal agencies visited dairy plant X. The plant purchased raw milk from over 59 different farms. Finished product was distributed in seven counties in eastern Pennsylvania as well as to Delaware and New Jersey. Dairy plant X was regularly inspected every 3 months by the Pennsylvania Department of Agriculture. The most recent inspection before the outbreak was in January 2000; no problems were reported.

According to our review of time and temperature pasteurization charts, pasteurization was adequate during the time of the outbreak. Our review of in-house microbial testing results from January 3 and April 17 identified 13 instances where the standard plate count was elevated and 9 instances where coliforms were elevated. The highest standard plate count was 120,000/mL on April 4, and the highest total coliform count was reported as >100/mL on April 14 and 17; both occurred in skim milk. According to the Pasteurized Milk Ordinance, the standard plate count should not exceed 20,000/mL, and the coliform count should not exceed 10/mL.

Inspectors from the Food and Drug Administration (FDA) found violations of sanitary standards that could have resulted in contamination of products after pasteurization. These violations included evidence of excessive condensation throughout the processing and packaging area. High humidity and excessive condensation could have produced droplets that fell into open containers. In addition, several machines leaked raw milk onto the floor, and raw skim milk was held in a silo at >10°C (standard: 7.2°C).

Sixty-six milk samples with production dates from April 3 to 20 were collected and tested by the Pennsylvania Department of Agriculture. None grew *Salmonella*; two grew *Escherichia coli*. None of 26 environmental samples collected April 28 grew *Salmonella*. All 172 finished milk samples collected in May and July were negative for *Salmonella* and had <1 μg/mL phosphatase, indicating all were adequately pasteurized.

A review of records at Dairy Plant X identified 14 employees who were absent between March 20 and April 20. Three (21%) had a gastrointestinal illness with onset March 20, March 26, and one unknown; a stool sample from one yielded the outbreak-related strain of *S.* Typhimurium. All reported drinking finished products produced at the plant in the 5 days before their illness onset.

## Conclusions

We describe a large, multistate outbreak of multidrug resistant *S.* Typhimurium infections linked to pasteurized milk. *Salmonella* likely contaminated the containers or milk contact surfaces after pasteurization because of environmental conditions in the plant, likely originating in *Salmonella*-contaminated raw milk. Two dairy cow isolates of *S.* Typhimurium obtained during the outbreak period were outbreak-related strains, which suggests that these strains were circulating in Pennsylvania dairy herds. Although federal agencies asked for access to the farms that provided the milk to the plant, these farms were not identified, which prevented further preplant investigation.

*S.* Typhimurium resistance type AKSSuT that caused this outbreak appears to be emerging and raises similar concerns to those that surround *S.* Typhimurium definitive type 104 (DT104) (*6*). Antimicrobial drugs are commonly used to treat persons with salmonellosis and can be life saving in severe infections. Antimicrobial resistance can limit treatment options, can contribute to treatment failure, and is associated with increased deaths (*7*).

The importance of pasteurized milk as a source of salmonellosis is largely unknown. We reviewed the published literature and identified 12 outbreaks in the United States between 1960 and 2000 that were associated with pasteurized milk (Table). Of the 12 outbreaks, seven were caused by contamination after pasteurization, and five were caused by *Salmonella*. Although published reports are relatively rare, outbreaks may not be recognized for several reasons. Milk is an extremely common exposure, which makes reporting exposure to milk likely, obscuring an association. Typhimurium is one of the most common serotypes of *Salmonella,* which make detecting outbreaks more difficult in the absence of subtyping. Several factors may enhance detection of pasteurized milk-associated outbreaks: a very focal illness event, illness caused by an unusual strain, a method for subtyping surveillance strains, heavy contamination of the product, and a local brand of milk.

**Table:** Review of pasteurized milk outbreaks in the United States, 1960–2000

Date	Location (ref)	Pathogen	Setting	Total no. ill (confirmed)	Mechanism of contamination
Nov 1966	Florida (*8*)	*Shigella flexneri* type 2	Community	97 (97)	After pasteurization
Jul–Aug 1975	Louisiana (*9*)	*Salmonella* Newport	Military base/community	49 (49)	Unknown
Sep–Oct 1976	New York (*10*)	*Yersinia enterocolitica* O:8	School	38 (38)	After pasteurization
Oct 1978	Arizona (*11*)	*S.* Typhimurium	Community	23 (23)	After pasteurization
Jun–Jul 1982	Tennessee, Arkansas, Mississippi (*12*)	*Y. enterocolitica* O:13, 18	Community	172 (172)	Unknown
Jun–Aug 1983	Massachusetts (*13*)	*Listeria monocytogenes* 4b; phage type 2425A	Community	49 (40)	Unknown
Apr 1984	Kentucky (*14*)	*S.* Typhimurium	Convent	16 (16)	Inadequate pasteurization
Mar–Apr 1985	Illinois (*1*)	*S.* Typhimurium	Community	>150,000 (>16,000)	After pasteurization
Mar–Apr 1986	Vermont (*15*)	*Campylobacter jejuni* O:2, 36	School	33 (8)	Inadequate pasteurization
Jul 1994	Illinois (*2*)	*L. monocytogenes* 1/2b	Picnic	45 (11)	After pasteurization
Oct 1995	Vermont, New Hampshire (*3*)	*Y. enterocolitica* O:8	Community	10 (10)	After pasteurization
Mar–Apr 2000	Pennsylvania, New Jersey	*S.* Typhimurium, phage type 21, R-type AKSSuT	Community	93 (93)	After pasteurization

The outbreak we report led to immediate changes in dairy plant X. The plant hired an outside consultant and addressed FDA’s immediate concerns. In addition, the Pennsylvania Department of Agriculture began to integrate plant employee training with routine inspections. And finally, as routine inspection regimens did not prevent the outbreak, the findings from this investigation prompted FDA to move up its scheduled review of the state milk regulatory program. Although the results of this review are not available to federal authorities or the public, Pennsylvania’s milk control program now satisfies all of the FDA criteria for certification.

Current milk standards are designed largely to safeguard against a failure or breakdown in the process of pasteurization. Our review of milk-borne outbreaks suggests that inadequate pasteurization is a relatively uncommon event compared to contamination after pasteurization. Additional regulatory emphasis on post pasteurization monitoring, such as coliform and standard plate count, may be needed for adequate protection.
